# Antimicrobial Efficacy of Essential Oils and Their Combination Against Microorganisms Associated With Postradiation Therapy in Patients With Head and Neck Cancer: An In Vitro Study

**DOI:** 10.7759/cureus.40768

**Published:** 2023-06-21

**Authors:** Mehul A Shah, Roopali M Sankeshwari, Anil V Ankola, Suneel Dodamani, Shivani Tendulkar, Sagar Jalihal, Atrey J Pai Khot, Anu Sara Varghese, Prajakta Chavan

**Affiliations:** 1 Department of Public Health Dentistry, KLE Vishwanath Katti Institute of Dental Sciences, KLE Academy of Higher Education and Research, Belagavi, IND; 2 Department of Microbiology, Basic Science Research Centre, KLE Academy of Higher Education and Research, Belagavi, IND; 3 Department of Public Health Dentistry, KLE Vishwanath Katti Institute of Dental Sciences, KLE Academy of Higher Education and Research, Belagavi, India, Belagavi, IND

**Keywords:** minimum inhibitory concentration (mic), mouth neoplasms, colony-forming units, essential oil mouth rinse, chemo-radio (chemoradiotherapy), antimicrobial effect

## Abstract

Background

Head and neck cancer ranks as the sixth most common cancer globally. Reduced saliva production brought on by postradiation therapy upsets the delicate balance between bacterial load and a weakened immune system. Oral hygiene is commonly neglected in patients who have undergone radiotherapy and they often develop dry mouth, mucositis due to radiation therapy, etc., as side effects. Despite being a part of the current standard, chlorhexidine carries numerous disadvantages such as taste alteration, teeth staining, and dry mouth. An extensive review of the literature demonstrates the antibacterial properties of essential oils (EOs) derived from plant materials, which may be able to prevent the development of such opportunistic microorganisms in the oral cavity.

Methodology

The cinnamon bark EO and Cajeput EO were procured and checked for their solubility. The final ratio at which the oils were found to be soluble was the 1:1 (w/v) ratio. The minimum inhibitory concentration (MIC) of cinnamon bark oil (*Cinnamomum verum*) and Cajeput oil (*Melaleuca leucadendron*) against *Staphylococcus aureus, Enterococcus faecalis, *and* Candida albicans* was determined by serial dilution method using Resazurin dye, and the minimum bactericidal concentration (MBC) was done by a spread plating method. The polyherbal mouthwash was subjected to cytotoxicity assay against human gingival fibroblasts. All the experiments were performed in triplicates.

Results

The overall results showed that cinnamon bark EO had the strongest efficacy against *S. aureus *(0.33 ± 0.14 mg/mL) and *E. faecalis* (0.41 ± 0.14 mg/mL), but not against *C. albicans *(2.85 ± 2.11 mg/mL). Cajeput EO showed the least efficacy against all the groups; whereas the combination of EOs proved to be the most efficacious and showed good antimicrobial activity against these most commonly encountered microorganisms in head and neck cancer postradiotherapy.

Conclusions

Cinnamon and Cajeput EOs in combination proved to be effective in this in vitro study against the most common microorganisms encountered in patients with head and neck cancer postradiotherapy and are comparable to 0.2% chlorhexidine.

## Introduction

Head and neck cancers rank sixth among all the cancers occurring worldwide. Each year, it causes around 600,000 new cases and around 350,000 fatalities globally [1]. According to the worldwide agency for cancer research, the number of cancer cases in India is expected to rise from one million in 2012 to more than 1.7 million by 2035 [2]. Despite many efficient treatment modalities for oral cancer, such as aciurgy, irradiation, chemo, or a combination of these, local and pervasive immunity decreases among the treated patients. An unbalanced oral environment may be the result of hyposalivation, swallowing issues, and differences in the quality, quantity, and complexity of oral bacteria [3]. Moreover, precancerous diseases and malignancies have been linked to increased levels and altered microbial composition in the oral cavity [4].

The majority of oral cavity organisms engage in commensalism, which means that both the host and the bacteria benefit biologically from one another [5]. The commensal populations do not cause harm and maintain a check on the pathogenic species by not allowing them to adhere to the mucosa. The bacteria become pathogenic only after they breach the barrier of the commensals, causing infection and disease [6]. But within this largely harmless flora, there are dangerous germs that can spread disease [5]. Even normally benign organisms can become aggressive in the presence of an immunocompromised state and cause a variety of issues, such as inflammation, degeneration, cancer, or temporary illnesses [5]. Firmicutes, Proteobacteria, Bacteroidetes, Actinobacteria, and Fusobacteria are the most prevalent and numerous phyla associated with oral neoplasm [5-7]. Streptococcus species are the most prevalent oral bacteria found in healthy persons, while anaerobic Prevotella, Veillonella, Neisseria, and Haemophilus are less common. Before radiotherapy, *Staphylococcus* species were shown to be significantly abundant in patients with oral cancer, while *Candida albicans*, *Klebsiella* species, and *Pediococcus* species were the important pathogens isolated from patients with cancer after radiotherapy [5-7]. 

Oral hygiene is frequently overlooked in patients who have endured resection for oral tumors and after radiotherapy. Sometimes, patients may be noncompliant or too unwell to implement conventional oral hygiene practices on their own and it may only be possible with the assistance of nursing staff, family members, and allied health professionals. Postradiation therapy causes decreased stimulated saliva production, which may cause an alteration in oral microflora, resulting in the disruption of the balance between bacterial load and a compromised immune status [8].

Following radiotherapy, the patient may develop drug-resistant opportunistic infections from oral cavity microorganisms like *C. albicans*, *Staphylococcus aureus*, and *Enterococcus faecalis*, which may lead to systemic problems and significant morbidity in immunocompromised people. *S. aureus* causes epidermal and soft tissue infections such as furuncles, abscesses (boils), and cellulitis. They are also responsible for serious infections such as bloodstream infections, pneumonia, or bone and joint infections. *E. faecalis* can cause septicemia, meningitis, urinary tract infections, and a multitude of other infections. It has been found to colonize dental implants to cause peri-implantitis. Candidiasis is a mycotic infection caused by a yeast (a type of fungus) called Candida - the most common of which is *C. albicans*. If the infection spreads or if it enters deep into the body (e.g., the bloodstream or internal organs like the kidney, heart, or brain) it causes systemic Candidemia, which may prove to be fatal [8].

A thorough literature review shows the antimicrobial activity of essential oils (EOs) obtained from plant materials, which have the potential to inhibit the growth of such opportunistic microorganisms in the oral cavity. Cinnamon bark EO and Cajeput EO have been recognized for their antimicrobial efficacy against various microorganisms, such as *S. aureus*, *Escherichia coli*, *Acinetobacter baumannii*, *E. faecalis*, *C. albicans*, and more [9]. Additionally, *Pseudomonas aeruginosa*, *Micrococcus luteus*, *S. aureus*, *Staphylococcus epidermidis*, *E. faecalis*, *Klebsiella* sp., *Staphylococcus capitis*, and others have shown susceptibility to Cajeput EO [9].

Oral hygiene is commonly neglected in patients who have undergone radiotherapy and they often develop dry mouth, mucositis due to radiation therapy, etc., as side effects. Chlorhexidine (CHX) has a broad spectrum of dose-dependent antibacterial action. Despite being a part of the current standard regime for its antimicrobial effect, CHX carries numerous disadvantages such as taste alteration, teeth staining, and dry mouth. Thus, it is imperative to develop other means of oral hygiene practices in such situations. Therefore, this study aimed to assess the efficacy of Cinnamon bark oil (*Cinnamomum verum*) and Cajeput oil (*Melaleuca leucadendron*) and their synergism against the most common opportunistic microorganisms seen in postradiation therapy in patients with head and neck cancer.

## Materials and methods

The cinnamon bark EO (*C. verum*) and Cajeput EO (*M. leucadendron*) were procured and authenticated from Nishant Aromas Private Limited. It was then deposited to the Department of Pharmacognosy for drug authorization and phytochemical screening. Ethical clearance for the study was obtained from the Institutional Review Board. The following microbiological strains were used in this study: *S. aureus* (Sa), MTCC 12598; *E. faecalis* (Ef), MTCC 35550; and *C. albicans* (Ca), MTCC 2091. The standard strains of these microorganisms were procured from the microbiological depository. They were revived and subcultured using Brain Heart Infusion broth (BHI), supplemented with horse serum, and maintained on selective blood agar plates at 37 °C under aerobic conditions for 24 to 48 hours for the growth of bacteria. Anaerobic cultures (facultative anaerobes like *E. faecalis*) were incubated in an anaerobic jar where a mixture of chemicals with cold catalase produced 80% nitrogen (N_2_), 10% carbon dioxide (CO_2_), and 10% hydrogen (H_2_). CO_2_ released from the reaction stimulated the growth of the anaerobes, which were incubated for 48 to 72 hours.

The cinnamon bark EO and Cajeput EO were procured and checked for their solubility in dimethyl sulfoxide (DMSO). The oils were found to be miscible with each other as well as with DMSO. The final ratio at which the oils were found to be soluble was 1:1 (i.e., EO DMSO). This ratio was used thereafter for carrying out the MIC. The preparation for two stock solutions of cinnamon EO and Cajeput EO with DMSO was in the ratio of 1:1; their combination was prepared in the ratio of 0.5:0.5:1, that is, cinnamon EO:Cajeput EO:DMSO. The concentration in the first well was taken as 500 mg/mL. Serial dilution was repeated up to 10-12 for the EOs to reach the concentration of 0.244 mg/mL.

To prepare 50 mL of the standard solution of 0.2% CHX solution, 10 mg of 100% CHX salt was mixed in 50 mL of distilled water. The solution was kept in Ultrasonic Bath Sonicator (Branson Bransonic® M Mechanical Bath 1800, Danbury, CT, USA) at the Basic Science Research Centre (BSRC) for two minutes to ensure proper dissolution of the CHX salt. A 100 µL of microbial strains were inoculated along with BHI broth without the EOs.

The minimum inhibitory concentration (MIC) of cinnamon bark oil (*C. verum*)and Cajeput EO (*M. leucadendron*) against *S. aureus*, *E. faecalis*, and *C. albicans* was determined by serial dilution method using Resazurin dye. The tests were conducted in triplicates.

Following Clinical and Laboratory Standards Institute (CLSI) guidelines, the broth dilution method was carried out in 96 multiwell microtiter plates (Tarsons® 980040 PS 96 F Wells Tissue Culture Plate-Sterile, Korea) containing eight rows and 12 columns. Each 96-well plate was used for one single microorganism. The first, second, and third rows were assigned for cinnamon EO, Cajeput EO, and their combination, respectively. Fourth was left unused. To the fifth row, broth and diluent were added to ensure that the diluent was not fatal for the microorganisms. The sixth row tested the efficacy of the positive control CHX against the microorganisms, whereas the seventh row comprised growth control, which had broth and microorganisms.

One hundred microliters of sterile nutrient broth were added to all the aforementioned wells. Different concentrations (first well - 500 mg/mL to 12th well - 0.244 mg/mL) of the test sample were transferred to the wells to attain the twofold serial dilutions up to 10 to 12. Finally, 100 µL of microbial inoculum was added to each well. Plates were prepared in triplicates for *S. aureus*, *E. faecalis*, and *C. albicans*. The 96-well microtiter plates were sealed and placed in the incubator set at 37 °C for 18-24 hours. After 24 hours, 10 µL of freshly prepared Resazurin dye (0.015%) was aseptically added to each well under the ultraviolet light laminar airflow. The plates were incubated for four hours. Any color change from blue/purple to pink/red indicated viable microorganisms (Resazurin reduced to Resorufin) and no change of Resazurin dye indicated inhibition of microbial growth. The lowest concentration at which the color change was observed was taken as the MIC value of the respective EO and their combination against Sa, Ef, and Ca.

To obtain a 100 mL agar mix, 5.2 g of BHI agar (HiMedia®, Mumbai, India) was dissolved in 100 mL of distilled water and mixed well until evenly dispersed. (As per the manufacturer’s instructions, 52 g of BHI agar was dissolved in 1,000 mL of sterile water.) It was autoclaved at 121 to 37 °C, at 15 pounds per square inch of pressure for 20 minutes, and then cooled. The medium was transferred to the culture plates before its solidification, which was autoclaved and dried to avoid contamination. These plates were used for the determination of minimum bactericidal concentration (MBC).

The MBC for all EOs was determined by the spread plating method. Before streaking, the 96-well plates containing the EOs were blended with a pipette, followed by transferring a full loop culture onto the BHI agar plates and streaked in a zig-zag fashion. The MBC streaked plates were incubated for 16 to 18 hours, and the temperature was set at 37 °C. The plates were taken out of the incubator and observed for bacterial growth. These observations were compared with a 96-well microtiter plate employed for MIC determination. The concentration at which the microorganisms were completely killed was taken as the MBC. All the tests were performed in triplicates for all microorganisms. 3-(4,5-Dimethylthiazol-2-yl)-2,5-diphenyltetrazolium bromide (MTT) assay was employed to assess cell proliferation and cytotoxicity. The cells were subjected to the extract at three distinct concentrations for 48 hours, and cell viability was determined using gingival fibroblast [10]. Further information on this assay can be found in a separate study conducted elsewhere [11].

Data obtained were entered in Microsoft Excel (2019) and analyzed using IBM Corp. Released 2012 IBM SPSS Statistics for Windows, Version 21 (IBM Corp., Armonk, NY, USA). The descriptive statistics were presented as mean ± standard deviation for continuous variables. The following univariate analysis was performed: Kruskal Wallis test followed by the Bonferroni posthoc test to compare the differences in the antibacterial activity of individual EOs, their combination, and 0.2% CHX. *P*-value ≤ 0.05 was considered statistically significant.

## Results

The experiments were performed in triplicates. The mean MIC values of cinnamon bark EO, Cajeput EO, their combination, and 0.2% CHX solution against *S. aureus* were 0.33 ± 0.14 mg/mL, 5.21 ± 2.26 mg/mL, 0.57 ± 0.37 mg/mL, and 6.51 ± 2.26 mg/mL respectively, which suggested that cinnamon bark EO is the most effective bactericidal against *S. aureus* when compared to Cajeput EO and combination of EOs (Table 1).

**Table 1:** Determination of MIC of cinnamon essential oil, Cajeput essential oil, and their combination, and 0.2% CHX hydrochloride tested against Staphylococcus aureus, Enterococcus faecalis, and Candida albicans. The results are shown as average values of triplicates in mg/mL (mean ± standard deviation). The statistical test applied was the Kruskal-Wallis test. Bonferroni post hoc test was applied. ^a^Gold standard of 0.2% CHX is not statistically significant. ^b,c^Significant difference found when compared to 0.2% CHX (in the same row). *P ≤ 0.05 was considered statistically significant. MIC, minimum inhibitory concentration; CHX, chlorhexidine hydrochloride solution

Test microorganisms	MIC (mg/mL)				Statistics	
	0.2% CHX	Cinnamon oil	Cajeput oil	Combination	*Z*-value	*P*-value
*Staphylococcus aureus*	6.51 ± 2.26^a^	0.33 ± 0.14^b^	5.21 ± 2.26^a^	0.57 ± 0.37^c^	9.039	0.029*
*Enterococcus faecalis*	15.63 ± 0.02^a^	0.41 ± 0.14^b^	2.12 ± 1.71^a^	0.49 ± 0.42^c^	8.484	0.037*
*Candida albicans*	10.42 ± 4.51^a^	1.46 ± 2.11^a^	1.95 ± 0.01^a^	3.26 ± 3.95^b^	6.297	0.098

**Figure 1:**
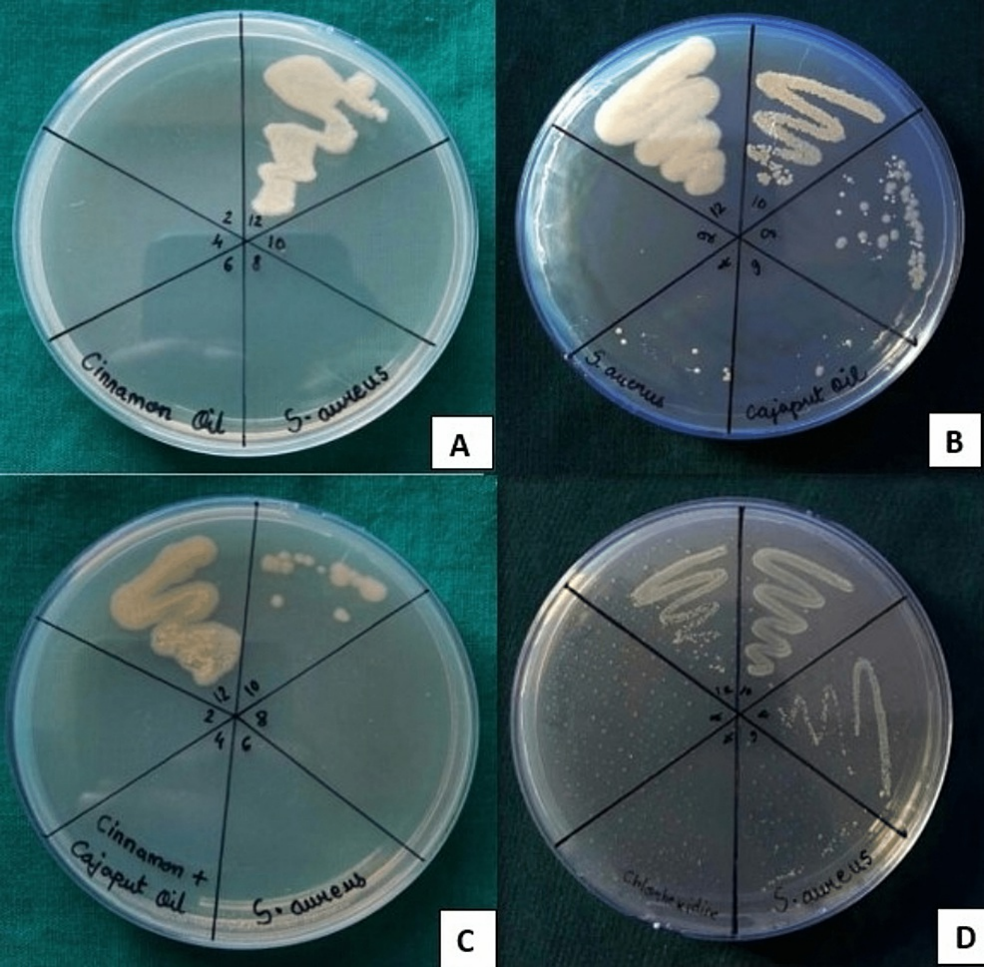
MBC of (A) cinnamon EO, (B) Cajeput EO, (C) their combination, and (D) 0.2% CHX against Staphylococcus aureus. (A) MBC of cinnamon bark EO against *S. aureus*. Visible growth was seen in the WN 12.  No growth was seen in the WN 10 (at the concentration of 0.488 mg/mL). This was taken as the MBC value of cinnamon EO against *S. aureus*. (B) MBC of Cajeput EO against *S. aureus*. Visible growth was seen in WN 12, WN 10, and WN 8.  No growth was seen in WN 6 (at the concentration of 15.624 mg/mL). This was taken as the MBC value of Cajeput EO against *S. aureus*. (C) Combination of EOs against *S. aureus*. Visible growth was seen in WN 12, and sparse growth was seen in WN 10. No growth was seen in WN 8 (at the concentration of 3.906 mg/mL). This was taken as the MBC value of the combination of EOs against *S. aureus*. (D) 0.2% CHX against *S. aureus*. Visible growth was seen in WN 12, WN 10, and WN 8, and sparse growth was seen in WN 6. No growth was seen in WN 4. This was taken as the MBC value of 0.2% CHX against *S. aureus*. WN, well number; MBC, minimum bactericidal concentration; EO, essential oil

The results obtained from MBC plates were in accordance with the MIC results. Cinnamon bark EO and combination EOs possessed comparable antimicrobial activity when compared to 0.2% CHX solution, but among the test groups, Cajeput EO was the least effective against *S. aureus *(Figure 1).

**Figure 2:**
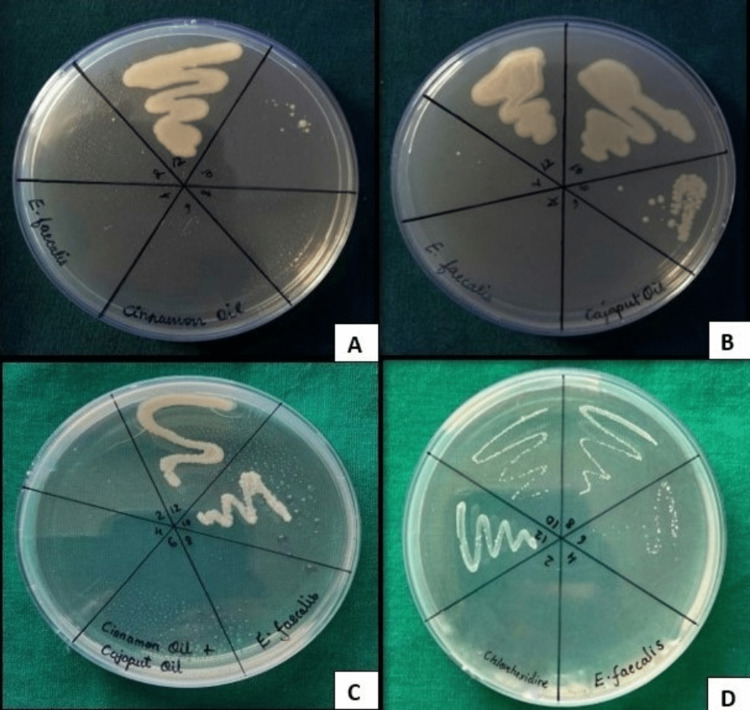
MBC of (A) cinnamon EO, (B) Cajeput EO, (C) their combination, and (D) 0.2% CHX against Enterococcus faecalis. (A) MBC of cinnamon bark EO against E. faecalis. Visible growth was seen in WN 12.  No growth was seen in WN 10 (at the concentration of 0.488 mg/mL). This was taken as the MBC value of cinnamon EO against *E. faecalis.* (B) MBC of Cajeput EO against *E. faecalis*. Visible growth was seen in WN 12, WN 10, and WN 8.  No growth was seen in WN 6 (at the concentration of 15.624 mg/mL). This was taken as the MBC value of Cajeput EO against *E. faecalis*. (C) MBC of a combination of EOs against *E. faecalis*. Visible growth was seen in WN 12 and WN 10. No growth was seen in WN 8 (at the concentration of 3.906 mg/mL). This was taken as the MBC value of the combination of EOs against *E. faecalis.* (D) 0.2% CHX against *E. faecalis*. Visible growth was seen in WN 12, WN 10, and WN 8, and sparse growth was seen in WN 6. No growth was seen in WN 4. This was taken as the MBC value of 0.2% CHX against *E. faecalis*. WN, well number;  MBC, minimum bactericidal concentration; EO, essential oil

The mean MIC values of cinnamon bark EO, Cajeput EO, their combination, and 0.2% CHX solution against *E. faecalis* were 0.41 ± 0.14, 2.12 ± 1.71, 0.49 ± 0.42, and 15.63 ± 0.02 mg/mL, respectively, which suggests that cinnamon bark EO is the most effective bactericidal against *E. faecalis* when compared to Cajeput EO and combination of EOs. The results obtained from MBC plates were in accordance with the MIC results (Figure 2). Cinnamon bark EO and combination EOs possess comparable antimicrobial activity when compared to 0.2% CHX solution, but among the test groups, Cajeput EO was the least effective against *E. faecalis*.

**Figure 3:**
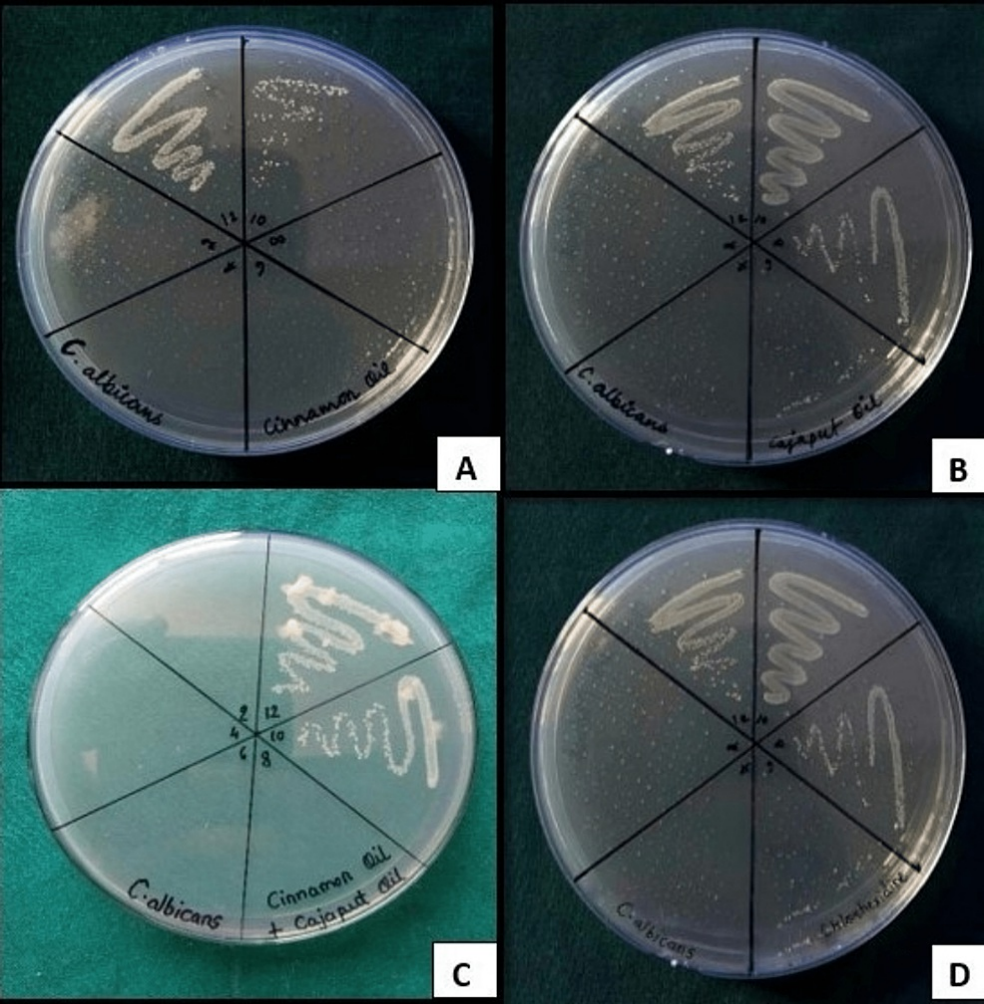
MBC of (A) cinnamon EO, (B) Cajeput EO, (C) their combination, and (D) 0.2% CHX against Candida albicans. (A) MBC of cinnamon bark EO against *C. albicans*. Visible growth was seen in WN 12 and WN 10.  No growth was seen in WN 8 (at the concentration of 3.906 mg/mL). This was taken as the MBC value of cinnamon EO against *C. albicans*. (B) MBC of Cajeput EO against *C. albicans*. Visible growth was seen in WN 12, WN 10, and WN 8.  No growth was seen in WN 6 (at the concentration of 15.624 mg/mL). This was taken as the MBC value of Cajeput EO against *C. albicans*. (C) MBC of a combination of EOs against *C. albicans*. Visible growth was seen in WN 12 and WN 10. No growth was seen in WN 8 (at the concentration of 3.906 mg/mL). This was taken as the MBC value of the combination of EOs against *C. albicans*. (D) 0.2% CHX against *C. albicans*. Visible growth was seen in WN 12, WN 10, and WN 8, and sparse growth was seen in WN 6. No growth was seen in WN 4. This was taken as the MBC value of 0.2% CHX against *C. albicans*. WN, well number;  MBC, minimum bactericidal concentration; EO, essential oil

The mean MIC values of cinnamon bark EO, Cajeput EO, their combination, and 0.2% CHX solution against *C. albicans* were 1.46 ± 2.11, 1.95 ± 0.01, 3.26 ± 3.95, and 10.42 ± 4.51 mg/mL, respectively, which suggests that cinnamon bark EO was the most effective fungicidal against *C. albicans* when compared to Cajeput EO and combination of EOs. The results obtained from MBC plates were in accordance with the MIC results (Figure 3), with the only difference being the MBC of the combination group (1.30 ± 0.56 mg/mL) was more efficacious when compared to the MIC (3.26 ± 3.95 mg/mL) results. Therefore, only the combination EOs possess comparable antimicrobial activity when compared to 0.2% CHX solution, but among the test groups, cinnamon bark EO and Cajeput EO were the least effective against *C. albicans* (Table 2).

**Table 2:** Determination of MBC of cinnamon essential oil, Cajeput essential oil, their combination, and 0.2% chlorhexidine gluconate against Staphylococcus aureus, Enterococcus faecalis, and Candida albicans. The results are shown as average values of triplicates in mg/mL (mean ± standard deviation). The statistical test applied was the Kruskal-Wallis test. The post hoc test applied was Bonferroni post hoc test. ^a^The gold standard of 0.2% CHX was not statistically significant. ^b,c^Significant differences when compared to 0.2% CHX (in the same row). *P ≤ 0.05 was considered statistically significant. MBC, minimum bactericidal concentration; CHX, chlorhexidine hydrochloride solution

Test microorganisms	MBC (mg/mL)				Statistics	
	0.2% CHX	Cinnamon oil	Cajeput oil	Combination	*Z*-value	*P*-value
*Staphylococcus aureus*	6.51 ± 2.26^a^	0.33 ± 0.14^b^	5.21 ± 2.26^a^	0.65 ± 0.28^c^	9.482	0.024*
*Enterococcus faecalis*	15.63 ± 0.02^a^	0.41 ± 0.14^b^	2.77 ± 1.97^a^	1.38 ± 0.99^c^	8.253	0.041*
*Candida albicans*	10.42 ± 4.51^a^	2.85 ± 2.11^a^	5.86 ± 3.38^a^	1.30 ± 0.56^b^	6.431	0.092

The highest concentration of the positive control (0.2% CHX solution) was required against *E. faecalis* (15.63 ± 0.02 mg/mL), whereas the lowest concentration was required against *S. aureus* (6.51 ± 2.26 mg/mL). The cinnamon bark EO was most effective against *S. aureus* and least against *C. albicans*. Similarly, Cajeput EO was most efficacious against *E. faecalis* and least against *C. albicans*. Finally, the combination EOs group was most efficacious against *S. aureus* and least with *E. faecalis*. The cell proliferation assay also showed that EOs maintained the cell viability of more than 75% of the gingival fibroblasts when compared to the gold standard CHX (Table 3).

**Table 3:** Mean of ODs of surviving cells against the combination mouthwash composed with cinnamon and Cajeput essential oils and chlorhexidine gluconate mouthwash at a wavelength of 570 nm. CHX, chlorhexidine gluconate mouthwash; Concentration 1 (least); Concentration 3 (most) - essential oil combination mouthwash; OD, optical density; IC50, half maximal inhibitory concentration

Compound	OD	Mean OD	Cell viability (%)	Results as observed	IC_50_ (μg)
Concentration 1 (1.383 mg/mL)	0.221	0.277	96.52	No lysis	No cell death at the highest concentration
	0.300				
	0.311				
Concentration 1 (1.383 mg/mL) × 2	0.340	0.274	95.36	No lysis	
	0.144				
	0.338				
Concentration 1 (1.383 mg/mL) × 3	0.201	0.272	94.55	No lysis	
	0.324				
	0.290				
CHX (0.2 mg/mL)	0.154	0.217	75.52	No lysis	
	0.264				
	0.233				

Therefore, the overall results showed that cinnamon bark EO had the strongest efficacy against *S. aureus* (0.33 ± 0.14 mg/mL) and *E. faecalis* (0.41 ± 0.14 mg/mL), but not against *C. albicans* (2.85 ± 2.11 mg/mL). Cajeput EO showed the least efficacy against all the groups, whereas the combination of EOs proved to be the most efficacious and showed good antimicrobial activity against these most commonly encountered microorganisms in patients with head and neck cancer postradiotherapy.

## Discussion

It is a well-known clinical reality that patients with oral cancer frequently have bad oral hygiene when they first come. According to studies, mouth microbiota may play a role in the emergence of cancer, and there may even be a causative relationship between oral microbes and cancer [12]. A common component of many chronic diseases, including cancer, is inflammation [11-12]. Inflammations brought on by infections are thought to contribute to the pathophysiology of about 15% to 20% of human malignancies [13].

Malignant and healthy oral mucosa appear to have different microbial populations. For instance, many upper gastrointestinal tract carcinomas have been linked to *Streptococcus anginosus* and *Treponema denticola* [11]. *S. anginosus* infection may be related to the development of head and neck squamous cell carcinoma, according to Tateda et al. [14]. Additionally, it should be remembered that the sampling site itself may affect the outcomes, making it challenging to conduct the best possible sampling in people with oral cancer [15].

A study conducted by Anjali et al. showed an increase in *Staphylococcus*, *Bacillus*, *Enterococcus*, *Pedicoccus*, and other germs such as *Klebsiella*, *Staphylococcus kloosii*, and *Staphylococcus hominis* following six months of radiation therapy [4]. When Kamath et al. examined how radiotherapy affected the oropharyngeal flora in head and neck cancer, they concluded that *S. aureus*, *Pseudomonas*, Bacteroides, and *C. albicans* increased, while *Streptococcus pneumoniae* considerably decreased after the end of irradiation [4].

A review conducted by Wińska et al. described the sources, chemical composition, and antimicrobial properties of various EOs. The main component of cinnamon EO is trans-cinnamaldehyde, which was also the primary constituent present in cinnamon bark EO, which was tested in this study [16]. O-methoxy-cinnamaldehyde, cinnamaldehyde, benzaldehyde, phenyl ethanol, borneol, eugenol, coumarin, and cinnamic acid are some of the other ingredients. Studies using EO from the cinnamic aldehyde-rich bark of *Cinnamomum zeylanicum* were conducted by Intorasoot et al. [17] and Lin-Feng et al. [18]. They demonstrated that cinnamon bark EO had a superior impact in comparison to other EOs (such as clove, lemongrass, tea tree, ginger, and basil), with excellent action against *S. aureus*, *E. coli*, and other pathogens. This study also showed excellent bactericidal activity of cinnamon bark EO against *S. aureus* at a pretty much lower MIC of 0.244 mg/mL [19].

One of the most characteristic components of Cajeput EO is 1,8-cineole (15%-60%) - with antimicrobial and anti-inflammatory activity; linalool - with antimicrobial, anti-inflammatory, analgesic, as well as antihyperalgesic activity; and terpinene-4-ol - with antibacterial, antiviral, and antifungal activities are also the active compounds present in commercially available EO used in this study. According to this study, Cajeput EO suppressed the growth of Gram-positive bacteria like *S. aureus* and *E. faecalis* at a concentration of 0.2% to 0.4%. It inhibits yeast like *C. albicans* at a 0.4% to 0.6% dosage. In this study, MIC of Cajeput EO was found to be 5.21 and 2.77 mg/mL against *S. aureus* and *E. faecalis*, respectively, whereas MIC was higher at a concentration of 5.67 mg/mL against *C. albicans*, which was by this study [5].

According to Metgud et al., greater numbers of bacterial and candidal colonies were found on the cancer site [5]. Although *Candida* sp., in particular *C. albicans*, has been linked to oesophageal and oral cancer, the pathogenic pathways and carcinogenic potential were unclear [16]. Hence, in this study, *C. albicans* was chosen as one of the most commonly found microorganisms in patients with oral cancer.

Several *C. zeylanicum* bark extracts were evaluated in vitro using the disk-diffusion method against *Klebsiella pneumonia* 13883, *Pseudomonas aeruginosa* American Type Culture Collection (ATCC) 27859, *S. aureus* 6538 P, *E. coli* (ATCC) 8739, and *E. faecalis* deoxycholate citrate Agar (DCA)-74 to determine their antibacterial effects. The EO whose principal component is cinnamaldehyde (97% by weight) showed antibacterial, antioxidant, and anti-inflammatory activity. The MIC value of 0.41 mg/mL, or 410 g/mL, was by the current investigation and ranged from 250 to 1,000 g/mL [20].

Although there is a lacuna in the literature regarding the cytotoxicity of Cajeput EO, a study done by Yen et al. revealed that as a food-grade EO, it can be used without any toxic effect on oral epithelial cells [21]. Although this study emphasizes the most commonly isolated microorganisms (*S. aureus*, *E. faecalis*, and *C. albicans*) in patients treated for head and neck cancer postradiotherapy; other numerous microorganisms that could reduce the life expectancy and lead to serious infections in such patients are not considered, which could be considered as the limitation of the study.

## Conclusions

Cinnamon and Cajeput EOs in combination possess comparable antimicrobial activity against *S. aureus*, *E. faecalis*, and *C. albicans* when compared to 0.2% CHX solution. The synergistic effect of the combination of cinnamon and Cajeput EOs significantly showed effectiveness against *C. albicans*, when compared to *S. aureus* and *E. faecalis*. The EOs and their combination were proven to be the least toxic when compared to CHX. Although the results of the present in vitro study are promising, extensive research is still required to prove the capability of such herbal products against other causative microorganisms present in patients treated for oral cancer postradiotherapy.
